# Supplementary figures and images for: SARS-CoV-2 vaccine uptake in a multi-ethnic UK healthcare workforce: A cross-sectional study
Source: PLoS Med. 2021 Nov 5;18(11):e1003823. doi: 10.1371/journal.pmed.1003823 (PMC8570522; doi:10.1371/journal.pmed.1003823)

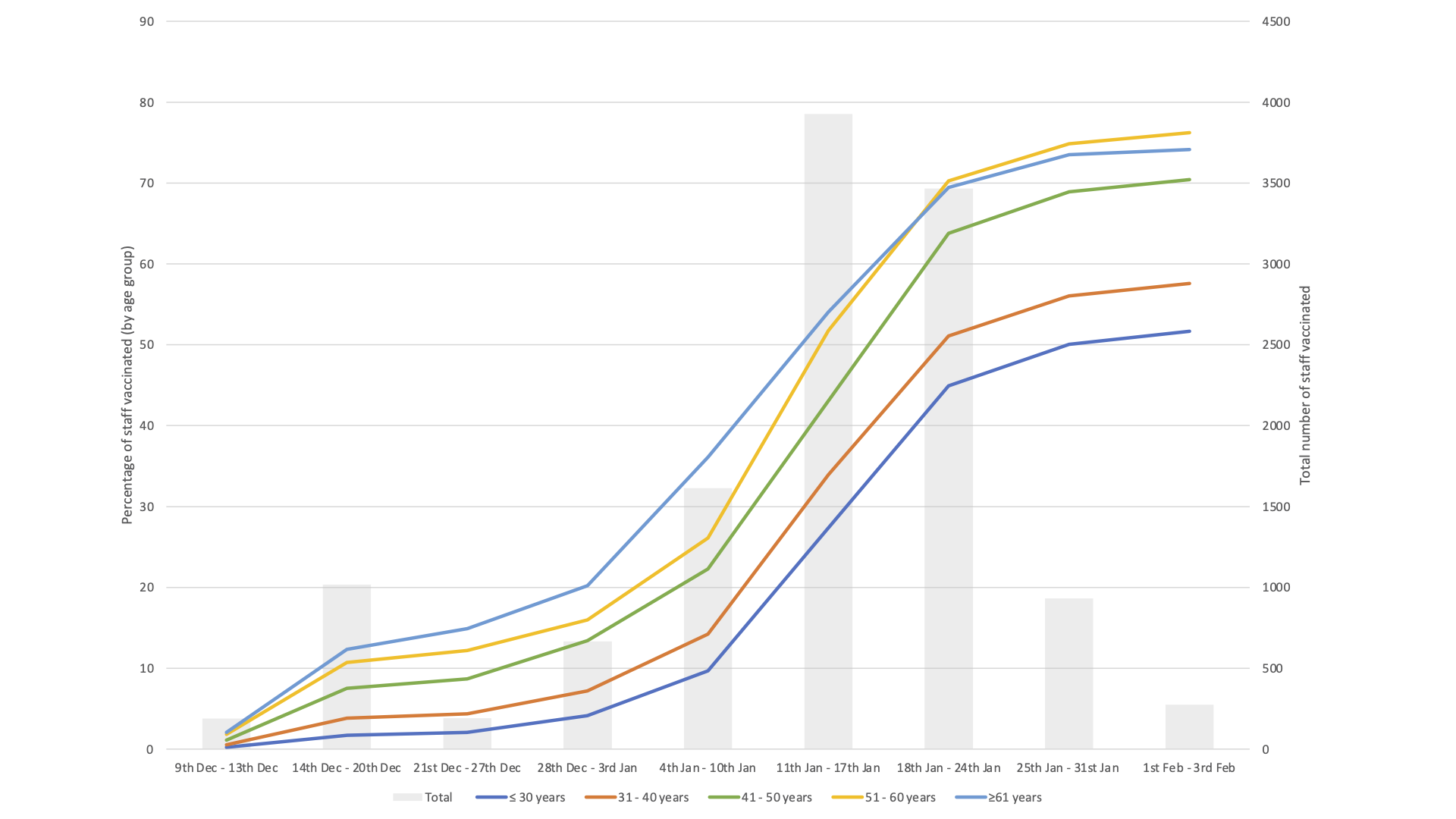

Supplement: S1 Fig — The figure shows the number of staff vaccinated (grey bars) and the cumulative percentage of the total number of staff of each age group vaccinated (coloured lines) each week since the start of the UHL vaccination programme. It should be noted that the first and last time points do not represent complete weeks. There was 1 missing value for date of vaccination, and this observation is excluded. (TIF) [file pmed.1003823.s002.tif]

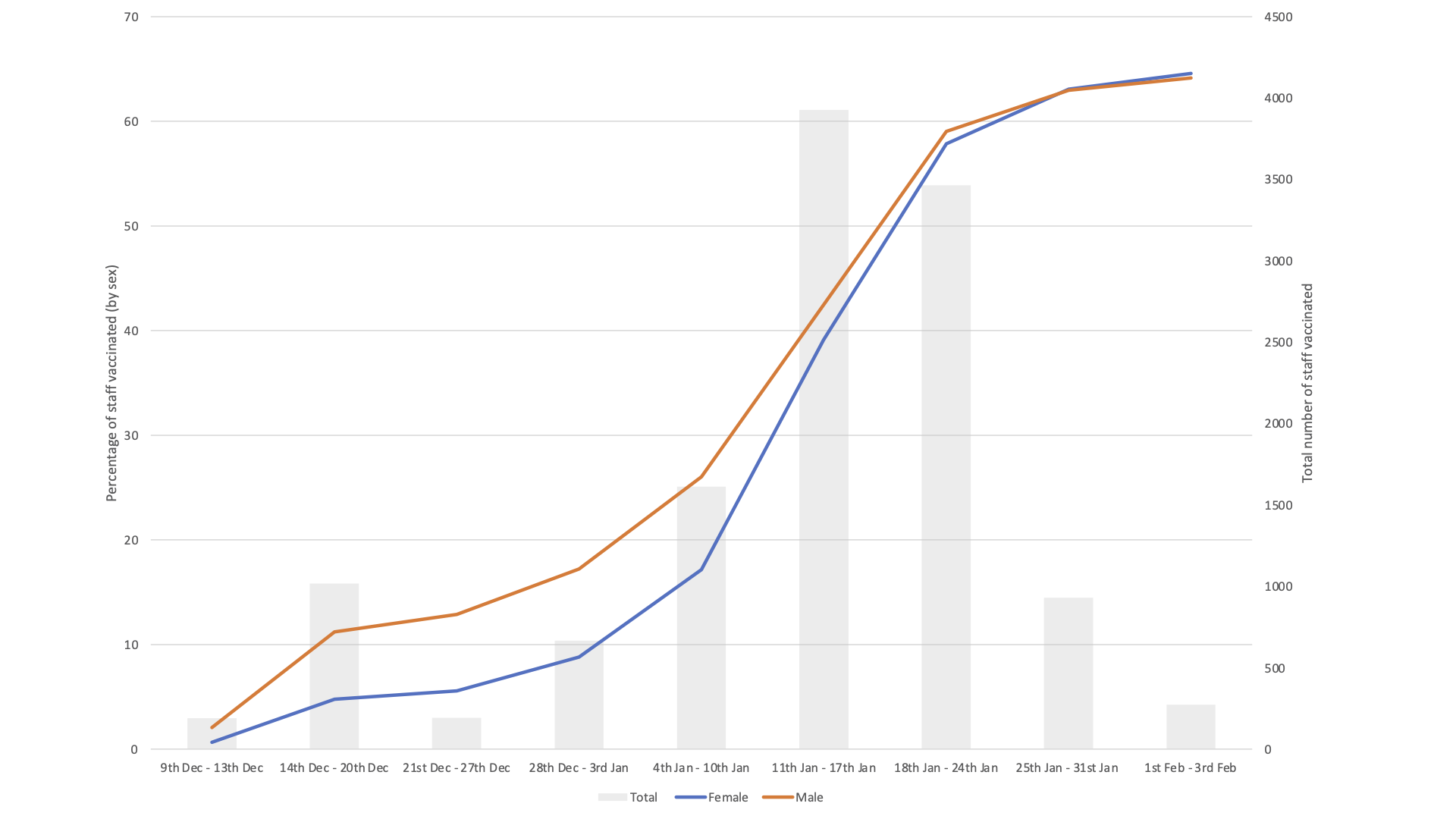

Supplement: S2 Fig — The figure shows the number of staff vaccinated (grey bars) and the cumulative percentage of the total number of staff of each sex vaccinated (coloured lines) each week since the start of the UHL vaccination programme. It should be noted that the first and last time points do not represent complete weeks. There was 1 missing value for date of vaccination, and this observation is excluded. (TIF) [file pmed.1003823.s003.tif]

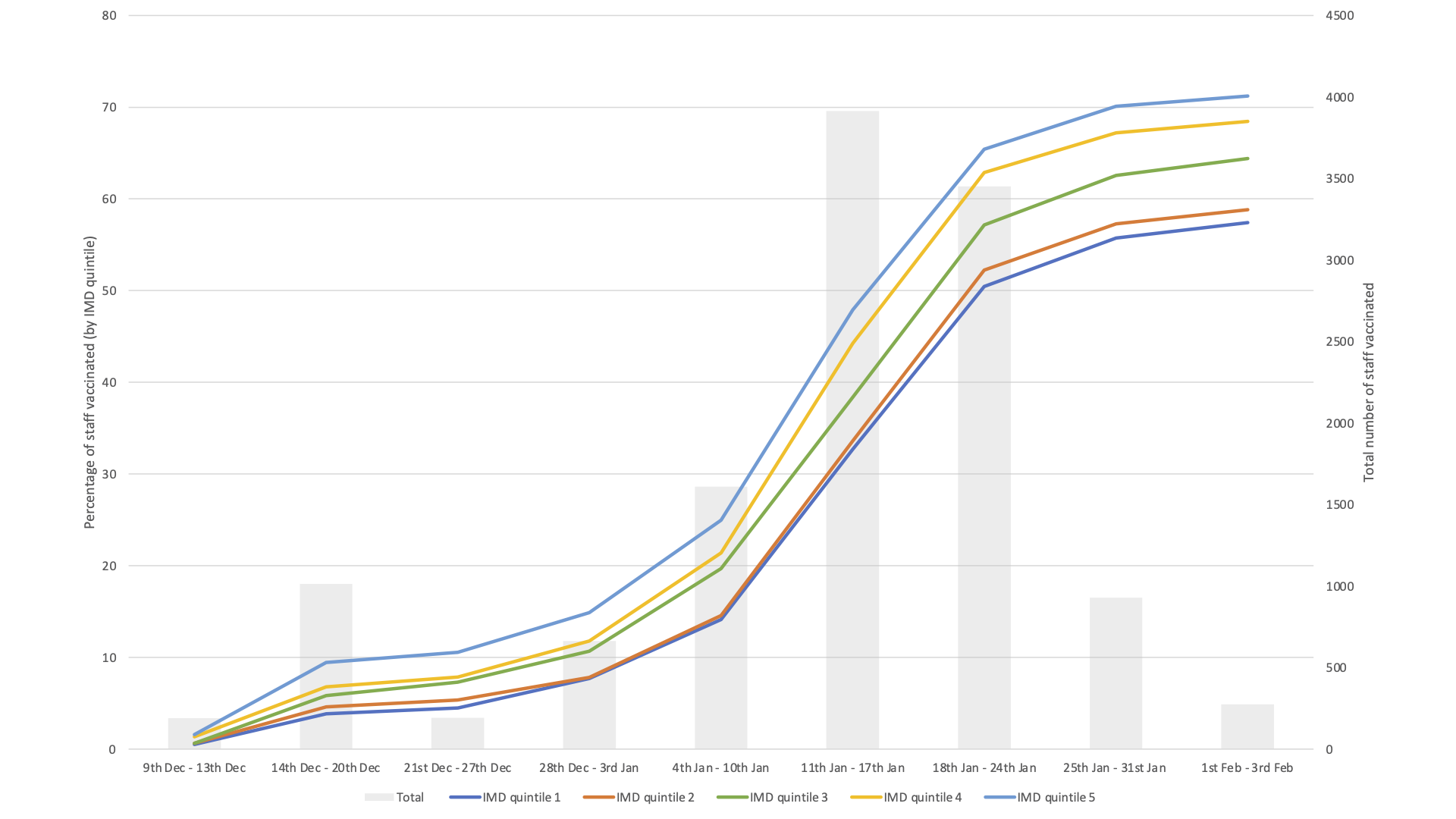

Supplement: S3 Fig — The figure shows the number of staff vaccinated (grey bars) and the cumulative percentage of the total number of staff of each Index of Multiple Deprivation quintile vaccinated (coloured lines) each week since the start of the UHL vaccination programme. It should be noted that the first and last time points do not represent complete weeks. There were 110 missing values for IMD and 1 missing value for date of vaccination, and these observations are excluded. (TIF) [file pmed.1003823.s004.tif]
