# Supplementary material for: SARS-CoV-2 vaccine uptake in a multi-ethnic UK healthcare workforce: A cross-sectional study
Source: PLoS Med. 2021 Nov 5;18(11):e1003823. doi: 10.1371/journal.pmed.1003823 (PMC8570522; doi:10.1371/journal.pmed.1003823)
Supplement: S1 Table — The table shows the categorisation of occupational role from the raw descriptions of occupational role in the Electronic Staff Record. (DOCX) [file pmed.1003823.s005.docx]

**S1 Table. Categorisation of occupational role**

| **OCCUPATIONAL ROLE (MANUSCRIPT)** | **OCCUPATIONAL ROLE (HEALTH RECORD)** |
| --- | --- |
| Doctors | "Consultant", "Foundation Year 1" ,"Core Trainee - Old Age", "Foundation Year 2", "Senior House Officer" "Specialist Registrar", "Specialty Doctor", "Specialty Registrar", Staff Grade", "Trust Grade Doctor - Career Grade level", "Trust Grade Doctor - Foundation Level", "Trust Grade Doctor - SHO Level"  "Trust Grade Doctor - Specialty Registrar", "Physician Associate", "Advanced Practitioner, "Associate Specialist (Closed to new entrants)", "Emergency Care Practitioner", "General Medical Practitioner", "Claims Doctor", "STR", "Specialist Registrar", "Trust Grade Doctor - Specialist Registrar", "Salaried General Practitioner", "Medical Student", “Hospital Practitioner”, “Clinical Assistant” |
| Nurses / HCAs | "Nurse Consultant", "Nurse Manager", "Nurse Qualified", "Nurse Qualified Hospital L1", "Nursery Nurse", "Nursing Associate", "Sister/Charge Nurse", "Staff Nurse", "Unqualified Nurse", "Health Care Assistant", "Healthcare Assistant", "Bank Midwife", "Midwife", "Midwife - Manager", "Midwife - Specialist Practitioner", "Modern Matron", "Enrolled Nurse", "Trainee Nursing Associate", "Trainee Practitioner", "Deputy Sister/Charge Nurse", "Assistant Practitioner Nursing", "Assistant/Associate Practitioner", "Assistant/Associate Practitioner Nursing", "Health Care Support Worker", "Director of Nursing" "Ward Sister/Charge Nurse" |
| Allied Health Professionals | "Occupational Therapist", "Occupational Therapist Manager", "Occupational Therapy Specialist Practitioner", "Physiotherapist", "Physiotherapist Manager", "Physiotherapist Specialist Practitioner", "Operating Department Practitioner", "Phlebotomist", "Pharmacist", "Pre-reg Pharmacist", “Student Technician – Pharmacy” "Radiographer - Diagnostic", "Radiographer - Diagnostic, Consultant", "Radiographer - Diagnostic, Manager", "Radiographer - Diagnostic, Specialist Practitioner", "Radiographer - Therapeutic", “Student Technician –Trauma and Orthopaedic surgery” |
| Administrative/Executive | "Secretary", "Manager", "Accountant", "Medical Secretary", "Apprentice", "Officer", "Clerical Worker", "Board Level Director", “Chair”, "Librarian", "Finance Director", "Other Executive Director", "Receptionist", "18 Week Pathway Co-ordinator", "Adviser", "Senior Manager", "Personal Assistant", "Analyst", "Systems Manager" |
| Healthcare Scientists | "Healthcare Science Assistant", "Healthcare Science Associate", "Healthcare Science Practitioner", "Healthcare Scientist", "Researcher", "Consultant Healthcare Scientist", "Biomedical Scientist”, "Trainee Healthcare Science Associate", "Trainee Healthcare Science Practitioner ", "Trainee Healthcare Scientist", "Specialist Healthcare Scientist", "Specialist Healthcare Science Practitioner", “Student Technician – Clinical Cytogenetics” |
| Estates | "Assistant", "Building Craftsperson", "Carpenter", "Cleaner", "Cook", "Driver", "Electrician", "Engineer", "Gardener/Groundsperson", "Housekeeper", "Maintenance Craftsperson", "Porter", "Supervisor", "Technician", "Ward Housekeeper" |
| Other | "Chaplain", "Helper/Assistant", "Interpreter", "Network Engineer" |
