# Supplementary material for: SARS-CoV-2 vaccine uptake in a multi-ethnic UK healthcare workforce: A cross-sectional study
Source: PLoS Med. 2021 Nov 5;18(11):e1003823. doi: 10.1371/journal.pmed.1003823 (PMC8570522; doi:10.1371/journal.pmed.1003823)
Supplement: S2 Table — The table shows the categorisation of ethnicity into the categorical variable used in the main analysis from the descriptions of ethnicity in the Electronic Staff Record. (DOCX) [file pmed.1003823.s006.docx]

**S2 Table. Categorisation of ethnicity**

| **ETHNICITY (MANUSCRIPT)** | **ETHNICITY (ELECTRONIC STAFF RECORD)** |
| --- | --- |
| White | A White – British, B White – Irish, C White - Any other White background |
| South Asian | "H Asian or Asian British - Indian","J Asian or Asian British - Pakistani", "L Asian or Asian British - Bangladeshi", "K Asian or Asian British - Bangladeshi","L Asian or Asian British - Any other Asian background" |
| Black | "M Black or Black British - Caribbean", "N Black or Black British - African", "P Black or Black British - Any other Black background" |
| Other | "D Mixed - White & Black Caribbean", "E Mixed - White & Black African", "F Mixed - White & Asian", "G Mixed - Any other mixed background”, "R Chinese" |
| Not Stated | “Z Not Stated” |
