# Supplementary material for: SARS-CoV-2 vaccine uptake in a multi-ethnic UK healthcare workforce: A cross-sectional study
Source: PLoS Med. 2021 Nov 5;18(11):e1003823. doi: 10.1371/journal.pmed.1003823 (PMC8570522; doi:10.1371/journal.pmed.1003823)
Supplement: S3 Table — The table shows the proportion of vaccinated and unvaccinated staff of each ethnic group. Classification of ethnic groups was based on the 18 Office for National Statistics categories. Small numbers in some of these groups necessitated collapsing certain categories. (DOCX) [file pmed.1003823.s007.docx]

**S3 Table. Vaccination status by ethnicity subcategory**

| **Ethnicity** | **Unvaccinated, n(%)** | **Vaccinated, n(%)** |
| --- | --- | --- |
| White British / Irish | 2794 (27.8) | 7262 (72.2) |
| Other White | 544 (38.1) | 885 (61.9) |
| Indian | 1477 (39.8) | 2239 (60.3) |
| Pakistani | 249 (56.9) | 189 (43.2) |
| Bangladeshi | 74 (63.3) | 43 (36.8) |
| Other South Asian | 220 (37.2) | 372 (62.8) |
| Black Caribbean | 137 (60.4) | 90 (39.7) |
| Black African | 641 (63.8) | 363 (36.2) |
| Other Black | 80 (63.5) | 46 (36.5) |
| Mixed White / Black (African or Caribbean) | 96 (47.8) | 105 (52.2) |
| Mixed White / Asian | 39 (42.9) | 52 (57.1) |
| Other Mixed | 45 (43.3) | 59 (56.7) |
| Chinese | 61 (40.4) | 90 (59.6) |
| Other | 188 (38.3) | 303 (61.7) |
| Not stated | 121 (40.2) | 180 (59.8) |
