# Supplementary material for: SARS-CoV-2 vaccine uptake in a multi-ethnic UK healthcare workforce: A cross-sectional study
Source: PLoS Med. 2021 Nov 5;18(11):e1003823. doi: 10.1371/journal.pmed.1003823 (PMC8570522; doi:10.1371/journal.pmed.1003823)
Supplement: S4 Table — The table shows a description of the cohort excluding those with locum or bank contracts by vaccination status. All data are presented as n (%). COVID-19, coronavirus disease 2019; HCA, healthcare assistant; IMD, Index of Multiple Deprivation; PCR, polymerase chain reaction; SARS-CoV-2, severe acute respiratory syndrome coronavirus 2. (DOCX) [file pmed.1003823.s008.docx]

| **Variable** | **Total**  n=16,433 | **Unvaccinated**  n=4917  (29.9%) | **Vaccinated**  n=11,516  (70.1%) |
| --- | --- | --- | --- |
| **Age (years)**  ≤30  31 – 40  41 – 50  51 – 60  ≥61 | 3490 (21.2)  4014 (24.4)  3873 (23.6)  3791 (23.1)  1265 (7.7) | 1430 (29.1)  1477 (30.0)  970 (19.7)  802 (16.3)  238 (4.8) | 2060 (17.9)  2537 (22.0)  2903 (25.2)  2989 (26.0)  1027 (8.9) |
| **Sex**  Female  Male | 12625 (76.8)  3808 (23.2) | 3866 (78.6)  1051 (21.4) | 8759 (76.1)  2757 (23.9) |
| **Ethnicity**  White  South Asian  Black  Other  Not stated | 10097 (61.4)  4203 (25.6)  1034 (6.3)  870 (5.3)  229 (1.4) | 2426 (49.3)  1536 (31.2)  583 (11.9)  297 (6.0)  75 (1.5) | 7671 (66.6)  2667 (23.2)  451 (3.9)  573 (5.0)  154 (1.3) |
| **IMD quintile**  5 (least deprived)  4  3  2  1 (most deprived)  Missing | 3998 (24.3)  3486 (21.2)  2850 (17.3)  3526 (21.5)  2516 (15.3)  57 (0.4) | 933 (19.0)  906 (18.4)  875 (17.8)  1254 (25.5)  931 (18.9)  18 (0.4) | 3065 (26.6)  2580 (22.4)  1975 (17.2)  2272 (19.7)  1585 (13.8)  39 (0.3) |
| **Occupation**  Doctor  Nurse / HCA  Allied Health Professional  Admin / executive  Healthcare Scientist  Estates / Facilities  Other | 2299 (14.0)  6669 (40.6)  1281 (7.8)  3209 (19.5)  806 (4.9)  1978 (12.0)  191 (1.2) | 704 (14.3)  2157 (43.9)  364 (7.4)  768 (15.6)  198 (4.0)  678 (13.8)  48 (1.0) | 1595 (13.9)  4512 (39.2)  917 (8.0)  2441 (21.2)  608 (5.3)  1300 (11.3)  143 (1.2) |
| **Previous SARS-CoV-2 serology**  Never tested  Negative  Positive | 5611 (34.1)  9669 (58.8)  1153 (7.0) | 2250 (45.8)  2363 (48.1)  304 (6.2) | 3361 (29.2)  7306 (63.4)  849 (7.4) |
| **Previous SARS-CoV-2 PCR**  Never tested  Negative  Positive | 12,765 (77.7)  2, 886(17.6)  782 (4.8) | 4018 (81.7)  638 (13.0)  261 (5.3) | 8747 (76.0)  2248 (19.5)  521 (4.5) |
| **Previous COVID-19 work absence**  No absence  Symptomatic  Household / test and trace contact  Pregnant | 9878 (60.1)  3698 (22.5)  2727 (16.6)  130 (0.8) | 2794 (56.8)  1221 (24.8)  796 (16.2)  106 (2.2) | 7084 (61.5)  2477 (21.5)  1931 (16.8)  24 (0.2) |

**S4 Table. Description of cohort excluding locum or bank staff**
