# Supplementary material for: SARS-CoV-2 vaccine uptake in a multi-ethnic UK healthcare workforce: A cross-sectional study
Source: PLoS Med. 2021 Nov 5;18(11):e1003823. doi: 10.1371/journal.pmed.1003823 (PMC8570522; doi:10.1371/journal.pmed.1003823)
Supplement: S5 Table — The table shows a description of medical staff by vaccination status. The right-hand columns exclude those with locum or bank contracts. *Values redacted due to the potential for identification. Definitions of roles: foundation year 1 doctor (FY1), the first year of a 2-year training programme for doctors who have just left medical school; foundation year 2 doctor (FY2), the second year of the aforementioned programme; senior house officer (SHO), a doctor in training who has completed the foundation programme and has entered a ‘core’ training programme such as core medical or core surgical training but has not yet entered speciality training; specialist registrar (SpR), a doctor in training who has entered a speciality training programme; consultant, a hospital doctor who has completed training; trust grade, a doctor who is not in a training programme but is employed by the trust for provision of clinical services (may be of varying grades); medical support staff, roles such as physicians associates and advanced practitioners; general practitioner (GP). (DOCX) [file pmed.1003823.s009.docx]

**S5 Table. Vaccine uptake in medical staff**

| **Grade of medical staff** | **Total cohort** | | **Locum and bank workers excluded** | |
| --- | --- | --- | --- | --- |
|  | **Unvaccinated, n(%)** | **Vaccinated, n(%)** | **Unvaccinated, n(%)** | **Vaccinated, n(%)** |
| **FY1** | 35 (30.4) | 80 (69.6) | 34 (29.8) | 80 (70.2) |
| **FY2** | 50 (37.9) | 82 (62.1) | 50 (37.9) | 82 (62.1) |
| **SHO/SpR** | 689 (57.5) | 509 (42.5) | 287 (39.5) | 439 (60.5) |
| **Consultant** | 164 (18.6) | 720 (81.5) | 154 (17.7) | 718 (82.3) |
| **Trust grade** | 168 (41.3) | 239 (58.7) | 138 (37.1) | 234 (62.9) |
| **Medical support staff** | 130 (68.1) | 61(31.9) | 28 (40.6) | 41 (59.4) |
| **GP** | 19 (90.5) | * | 13 (92.9) | * |
| **Medical Student** | 25 (47.2) | 28 (52.8) | 0 | 0 |
