# Supplementary material for: SARS-CoV-2 vaccine uptake in a multi-ethnic UK healthcare workforce: A cross-sectional study
Source: PLoS Med. 2021 Nov 5;18(11):e1003823. doi: 10.1371/journal.pmed.1003823 (PMC8570522; doi:10.1371/journal.pmed.1003823)
Supplement: S6 Table — The table shows unadjusted and adjusted ORs for the association of covariates with the outcome of SARS-CoV-2 vaccine uptake. aOR, adjusted odds ratio (adjusted for all variables in the table); COVID-19, coronavirus disease 2019; HCA, healthcare assistant; IMD, Index of Multiple Deprivation; OR, odds ratio; PCR, polymerase chain reaction; SARS-CoV-2, severe acute respiratory syndrome coronavirus 2. (DOCX) [file pmed.1003823.s010.docx]

| Variable | N vaccinated / N total (%)  11,516 / 16,433 (70.1%) | OR (95% CI) | P value | aOR (95% CI) | P value |
| --- | --- | --- | --- | --- | --- |
| **Age (years)**  ≤30  31 – 40  41 – 50  51 – 60  ≥61 | 2060 / 3490 (59.0)  2537 / 4014 (63.2)  2903 / 3873 (75.0)  2989 / 3791 (78.8)  1027 / 1265 (81.2) | 0.48 (0.44 – 0.53)  0.57 (0.52 – 0.63)  Reference  1.25 (1.12 – 1.39)  1.44 (1.23 – 1.69) | <0.001  <0.001  <0.001  <0.001  <0.001 | 0.52 (0.47 – 0.58)  0.64 (0.58 – 0.71)  Reference  1.13 (1.01 – 1.26)  1.40 (1.19 – 1.65) | <0.001  <0.001  -  0.04  0.02 |
| **Sex**  Female  Male | 8759 / 12625 (69.4)  2757 / 3808 (72.4) | Reference  1.16 (1.07 – 1.25) | -  <0.001 | Reference  1.31 (1.20 – 1.45) | -  <0.001 |
| **Ethnicity**  White  South Asian  Black  Other  Not stated | 7671 / 10097 (76.0)  2667 / 4203 (63.5)  451 / 1034 (43.6)  573 / 870 (65.9)  154 / 301 (67.3) | Reference  0.55 (0.51 – 0.59)  0.24 (0.21 – 0.28)  0.61 (0.53 – 0.71)  0.65 (0.49 – 0.86) | -  <0.001  <0.001  <0.001  0.002 | Reference  0.63 (0.58 – 0.68)  0.29 (0.25 – 0.33)  0.69 (0.59 – 0.81)  0.58 (0.43 – 0.77) | -  <0.001  <0.001  <0.001  <0.001 |
| **IMD quintile**  5 (least deprived)  4  3  2  1 (most deprived) | 3065 / 3998 (76.7)  2580 / 3486 (74.0)  1975 / 2850 (69.3)  2272 / 3526 (64.4)  1585 / 2516 (63.0) | Reference  0.87 (0.78 – 0.96)  0.69 (0.62 – 0.77)  0.55 (0.50 – 0.61)  0.52 (0.47 – 0.58) | -  0.005  <0.001  <0.001  <0.001 | Reference  0.90 (0.81 – 1.01)  0.81 (0.72 – 0.90)  0.79 (0.71 – 0.88)  0.76 (0.67 – 0.86) | -  0.08  <0.001  <0.001  <0.001 |
| **Occupation**  Doctor  Nurse / HCA  Allied Health Professional  Admin / executive  Healthcare Scientist  Estates / Facilities  Other | 1595 / 2299 (69.4)  4512 / 6669 (67.7)  917 / 1281 (71.6)  2441 / 3209 (76.1)  608 / 806 (75.4)  1300 / 1978 (65.7)  143 / 191 (74.9) | Reference  0.92 (0.83 – 1.02)  1.11 (0.96 – 1.29)  1.40 (1.24 – 1.58)  1.36 (1.13 – 1.63)  0.85 (0.74 – 0.96)  1.31 (0.94 – 1.85) | -  0.13  0.17  <0.001  0.001  0.01  0.11 | Reference  0.85 (0.76 – 0.96)  1.06 (0.90 – 1.25)  1.09 (0.95 – 1.25)  1.29 (1.06 – 1.57)  0.66 (0.57 – 0.76)  1.10 (0.77 – 1.57) | -  0.009  0.47  0.20  0.01  <0.001  0.60 |
| **Previous SARS-CoV-2 serology**  Negative  Never tested  Positive | 7306 / 10314 (75.6)  3361 / 5611 (59.9)  849 / 1153 (73.6) | Reference  0.48 (0.45 – 0.52)  0.90 (0.79 – 1.04) | -  <0.001  0.15 | Reference  0.56 (0.51 – 0.60)  1.12 (0.97 – 1.29) | -  <0.001  0.14 |
| **Previous SARS-CoV-2 PCR**  Negative  Never tested  Positive | 2248 / 2886 (77.9)  8747 / 12765 (62.3)  521 / 782 (66.6) | Reference  0.62 (0.56 – 0.68)  0.57 (0.48 – 0.67) | -  <0.001  <0.001 | Reference  0.70 (0.63 – 0.78)  0.71 (0.59 – 0.85) | -  <0.001  <0.001 |
| **Previous COVID-19 related work absence**  No absence  Symptomatic  Household / test and trace contact  Pregnant | 7084 / 9878 (71.7)  2477 / 3698 (67.0)  1931 / 2727 (70.8)  24 / 130 (18.5) | Reference  0.80 (0.74 – 0.87)  0.96 (0.87 – 1.05)  0.09 (0.06 – 0.14) | -  <0.001  0.35  <0.001 | Reference  0.77 (0.71 – 0.85)  0.94 (0.85 – 1.04)  0.15 (0.10 – 0.24) | -  <0.001  0.22  <0.001 |

**S6 Table. Univariable and multivariable analysis of factors associated with SARS-CoV-2 vaccine uptake excluding those with locum or bank contracts**

aOR – adjusted odds ratio (adjusted for all variables in the table), HCA – healthcare assistant, IMD – index of multiple deprivation, OR – odds ratio, PCR – polymerase chain reaction, SARS-CoV-2 – severe acute respiratory syndrome coronavirus-2, COVID-19 – coronavirus disease 2019
