# Supplementary material for: SARS-CoV-2 vaccine uptake in a multi-ethnic UK healthcare workforce: A cross-sectional study
Source: PLoS Med. 2021 Nov 5;18(11):e1003823. doi: 10.1371/journal.pmed.1003823 (PMC8570522; doi:10.1371/journal.pmed.1003823)
Supplement: Attachment — Submitted filename: UHL vaccine - response to reviewers 18.08.21.docx [file pmed.1003823.s011.docx]

**Corresponding Author**

Dr. Manish Pareek,

Associate Clinical Professor in Infectious Diseases,

Department of Respiratory Sciences,

Maurice Shock Medical Sciences Building,

University of Leicester, University Road,

Leicester, LE1 9HN, UK

Email: [mp426@le.ac.uk](mailto:mp426@le.ac.uk)

Telephone: 0116 2586952

Dr Beryne Odeny

Associate Editor

*PLOS Medicine*

18^th^ August, 2021

Dear Dr Odeny,

**Authors’ response to reviews**

**Association of demographic and occupational factors with SARS-CoV-2 vaccine uptake in a multi-ethnic UK healthcare workforce: a rapid real-world analysis" (PMEDICINE-D-21-00819R1)**

**Now retitled (at editor’s request): Factors associated with SARS-CoV-2 vaccine uptake in a multi-ethnic UK healthcare workforce: A cross-sectional study**

We thank you for recognising the value of our work and for inviting us to submit a revised version of the above manuscript.

We are extremely grateful for the insightful comments made by the Editor and the reviewers and by incorporating their recommendations we have significantly improved the manuscript. We trust this will now prove suitable for publication.

Please find attached a detailed, point-by-point, response to the comments and suggestions made by the Editor and the reviewers.

Lastly, given the public health implications of our results, we would be grateful if the reviews of the revised manuscript could be fast-tracked.

Yours sincerely

**Dr. Manish Pareek**

Associate Clinical Professor in Infectious Diseases

**Response to Editor’s comments**

**Comment 1. Thank you very much for submitting your manuscript "Association of demographic and occupational factors with SARS-CoV-2 vaccine uptake in a multi-ethnic UK healthcare workforce: a rapid real-world analysis" (PMEDICINE-D-21-00819R1) for consideration at PLOS Medicine.**

*Response: Thank you for allowing us to submit a revised version of the manuscript.*

**Comment 2. Your paper was evaluated by a senior editor and discussed among all the editors here. It was also discussed with an academic editor with relevant expertise, and sent to independent reviewers, including a statistical reviewer.**

*Response: We are very grateful for the comments and suggestions that have come from such a thorough review both from your editorial team and the reviewers. We believe we have significantly improved the manuscript by incorporating their suggestions.*

**Comment 3. The reviews are appended at the bottom of this email and any accompanying reviewer attachments can be seen via the link below:**

*Response: Thank you for collating the reviews, we have considered them carefully and our point-by-point responses can be found attached. There do not seem to be any reviewer attachments.*

**Comment 4. In light of these reviews, I am afraid that we will not be able to accept the manuscript for publication in the journal in its current form, but we would like to consider a revised version that addresses the reviewers' and editors' comments.**

*Response: . We are very grateful for the opportunity to submit a revised manuscript*

**Comment 5: Obviously we cannot make any decision about publication until we have seen the revised manuscript and your response, and we plan to seek re-review by one or more of the reviewers.**

*Response: We accept that you cannot make decisions about publication until you have seen the revised manuscript and our response to the editorial and reviewer comments. We believe we have addressed all comments and hope that our revised manuscript will prove suitable for publication in PLOS Medicine.*

**Comment 6: In revising the manuscript for further consideration, your revisions should address the specific points made by each reviewer and the editors. Please also check the guidelines for revised papers at [hyperlink to guidelines] for any that apply to your paper.**

*Response: We have provided point-by-point responses to each of the editorial and reviewer comments and have checked the guidelines for revised papers.*

**Comment 7. In your rebuttal letter you should indicate your response to the reviewers' and editors' comments, the changes you have made in the manuscript, and include either an excerpt of the revised text or the location (eg: page and line number) where each change can be found.**

*Response: This rebuttal letter provides a response to each of the reviewers’ and editors’ comments highlighting where changes have been made in the manuscript by providing excerpts of the revised text.*

**Comment 8. Please submit a clean version of the paper as the main article file; a version with changes marked should be uploaded as a marked up manuscript.**

*Response: We have submitted both a clean and tracked version of the manuscript alongside this letter.*

**Comment 9. In addition, we request that you upload any figures associated with your paper as individual TIF or EPS files with 300dpi resolution at resubmission; please read our figure guidelines for more information on our requirements: [hyperlink to guidelines].**

*Response: We have uploaded our figures as per the specifications outlined above and consulted the relevant guidelines.*

**While revising your submission, please upload your figure files to the PACE digital diagnostic tool, [hyperlink to tool]. PACE helps ensure that figures meet PLOS requirements. To use PACE, you must first register as a user. Then, login and navigate to the UPLOAD tab, where you will find detailed instructions on how to use the tool. If you encounter any issues or have any questions when using PACE, please email us at** [**PLOSMedicine@plos.org**](mailto:PLOSMedicine@plos.org)**.**

*Response: We have uploaded our figure files to the PACE tool.*

**Comment 10. We expect to receive your revised manuscript by Aug 25 2021 11:59PM. Please email us (**[**plosmedicine@plos.org**](mailto:plosmedicine@plos.org)**) if you have any questions or concerns.**

*Response: We have uploaded our manuscript and the other required files before the deadline given above.*

**Comment 11. ***Please note while forming your response, if your article is accepted, you may have the opportunity to make the peer review history publicly available. The record will include editor decision letters (with reviews) and your responses to reviewer comments. If eligible, we will contact you to opt in or out.*****

*Response: If our article is accepted then we will await further instruction as to whether we will be offered the opportunity to publish the peer review history.*

**Comment 12. We ask every co-author listed on the manuscript to fill in a contributing author statement, making sure to declare all competing interests. If any of the co-authors have not filled in the statement, we will remind them to do so when the paper is revised.**

*Response: We believe all co-authors have filled in the statement, however, please let us know if this is not the case.*

**Comment 13. If all statements are not completed in a timely fashion this could hold up the re-review process.**

*Response: We believe all co-authors have filled in the statement so hopefully this will not delay the re-review process.*

**Comment 14. If new competing interests are declared later in the revision process, this may also hold up the submission.**

*Response: We believe all co-authors have filled in the statement. The competing interests statement in the original manuscript has not changed and, therefore, we hope this does not hold up the submission.*

**Comment 15. Should there be a problem getting one of your co-authors to fill in a statement we will be in contact.**

*Response: We believe all co-authors have filled in the statement. Please let us know if this is not the case.*

**Comment 16. YOU MUST NOT ADD OR REMOVE AUTHORS UNLESS YOU HAVE ALERTED THE EDITOR HANDLING THE MANUSCRIPT TO THE CHANGE AND THEY SPECIFICALLY HAVE AGREED TO IT.**

*Response: We have not added or removed any authors.*

**Comment 17. You can see our competing interests policy here: [hyperlink to policy]**

*Response: Thank you. We have consulted the policy.*

**Comment 18. Please use the following link to submit the revised manuscript:

[hyperlink to online submission portal]

Your article can be found in the "Submissions Needing Revision" folder.**

*Response: Thank you for these instructions.*

**Comment 19. To enhance the reproducibility of your results, we recommend that you deposit your laboratory protocols in protocols.io, where a protocol can be assigned its own identifier (DOI) such that it can be cited independently in the future.**

*Response: As our work describes an observational study using routinely collected data, there are no laboratory protocols associated with the work.*

**Comment 20. Additionally, PLOS ONE offers an option to publish peer-reviewed clinical study protocols. Read more information on sharing protocols at [hyperlink to information on sharing protocols]**

*Response: Our study represents an analysis of routinely collected data. We do not feel such a study warrants separate publication of a study protocol. Instead, we feel that an accurate and detailed description of methodological considerations is given in the manuscript.*

**Comment 21. Please ensure that the paper adheres to the PLOS Data Availability Policy (see [hyperlink to PLOS data availability policy]) which requires that all data underlying the study's findings be provided in a repository or as Supporting Information.**

*Response: In accordance with the PLOS data availability policy we are unable to make the data publicly available. This is because our analysis involves demographic data concerning employees at a named hospital trust. These data, in combination, could be used to identify individuals in the analysis.*

*These data were extracted under Caldicott guardian approval for the purposes of an audit aimed at establishing vaccine uptake in the trust. As part of this undertaking we are not to further routinely share the data. Parties interested in obtaining the original dataset should contact the corresponding author and requests will be dealt with on a case-by-case basis after discussion with the Caldicott guardian.*

*To make this clear in the manuscript we have now included a data sharing statement which reads:*

*The cohort was extracted under Caldicott Guardian approval for a specific purpose and as part of our undertaking with them we are not to further routinely share this data. The data are held in an institutional repository. Interested parties, with appropriate approvals, can apply for data access by contacting Tim Skelton – R&I Chief Operating Officer at UHL (tim.skelton@uhl-tr.nhs.uk). Reasonable requests will be assessed on a case-by-case basis in discussion with the Caldicott Guardian.*

**Comment 22. For data residing with a third party, authors are required to provide instructions with contact information for obtaining the data.**

*Response: . Please see our response to comment 21 above. The data are held in an institutional repository. Interested parties should contact Tim Skelton using the email address provided. We have included a data sharing statement to this effect within the manuscript.*

**Comment 23. PLOS journals do not allow statements supported by "data not shown" or "unpublished results." For such statements, authors must provide supporting data or cite public sources that include it*.***

*Response: Thank you. We do not include any such statements in the manuscript.*

**Comment 24. We look forward to receiving your revised manuscript.**

*Response: Thank you. We hope this revised article will now prove suitable for publication.*

**Comment 25. Please revise your title according to PLOS Medicine's style. Your title must be nondeclarative and not a question. It should begin with main concept if possible. Please place the study design (e.g., "A cross-sectional study") in the subtitle (i.e., after a colon). For example, “Factors associated with SARS-CoV-2 vaccine uptake in a multi-ethnic UK healthcare workforce: A cross-sectional study”**

*Response: We have changed the title to your suggestion. The title now reads:*

*“Factors associated with SARS-CoV-2 vaccine uptake in a multi-ethnic UK healthcare workforce: A cross-sectional study”*

**Comment 26.  The Data Availability Statement (DAS) requires revision. For each data source used in your study:
a) If the data are freely or publicly available, note this and state the location of the data: within the paper, in Supporting Information files, or in a public repository (include the DOI or accession number).
b) If the data are owned by a third party but freely available upon request, please note this and state the owner of the data set and contact information for data requests (web or email address). Note that a study author cannot be the contact person for the data.
c) If the data are not freely available, please describe briefly the ethical, legal, or contractual restriction that prevents you from sharing it. Please also include an appropriate contact (web or email address) for inquiries (again, this cannot be a study author).**

*Response: In accordance with the PLOS data availability policy we are unable to make the data publicly available. This is because our analysis involves demographic data relating to employees at a named hospital trust. These data, in combination, could be used to identify individual healthcare workers included in our analysis.*

*These data were extracted under Caldicott guardian approval for the purposes of an audit aimed at establishing vaccine uptake in the trust. As part of this undertaking we are not to further routinely share the data. Parties interested in obtaining the original dataset should contact Tim Skelton (using the details provided in the manuscript) and requests will be dealt with on a case-by-case basis after discussion with the Caldicott guardian.*

*To make this clear in the manuscript we have now included a data sharing statement which reads:*

*“The cohort was extracted under Caldicott Guardian approval for a specific purpose and as part of our undertaking with them we are not to further routinely share this data. The data are held in an institutional repository. Interested parties, with appropriate approvals, can apply for data access by contacting Tim Skelton – R&I Chief Operating Officer at UHL (tim.skelton@uhl-tr.nhs.uk). Reasonable requests will be assessed on a case-by-case basis in discussion with the Caldicott Guardian.”*

**Comment 27. Abstract: Please structure your abstract using the PLOS Medicine headings (Background, Methods and Findings, Conclusions).**

*Response: We have now structured the abstract accordingly.*

**Comment 28. Abstract: Please combine the Methods and Findings sections into one section, “Methods and findings”.**

*Response: We have now combined the methods and findings section into one section.*

**Comment 29. Abstract: Please ensure that all numbers presented in the abstract are present and identical to numbers presented in the main manuscript text.**

*Response: We have checked that the numbers presented in the abstract align with the main manuscript text.*

**Comment 30. Abstract: Please include the actual amounts or percentages of relevant outcomes, not just odds ratios.**

*Response: We have included percentages for each of the observations where an odds ratio is presented. We have not included odds ratios and percentages for all observations mentioned in the abstract as it was felt this would make the text difficult to read. However, we would be happy to add these in at the editor’s discretion.*

**Comment 31. Abstract: Please include the important dependent variables that are adjusted for in the analyses.**

*Response: We have included all the variables that were adjusted for in the logistic regression analysis. The text now reads:*

*“After adjustment for age, sex, ethnicity, deprivation, occupation, SARS-CoV-2 serology/PCR results and COVID-19 related work absences, factors found to be negatively associated with vaccine uptake were; younger age, female sex, increasing deprivation, pregnancy and belonging to any non-White ethnic group”*

**Comment 32. Abstract: Please include p values in addition to 95% CIs.**

*Response: Where 95% CIs are presented, we have now also presented a p value.*

**Comment 33. Abstract: In the last sentence of the Abstract Methods and Findings section, please describe the main limitation(s) of the study's methodology.**

*Response: We have now included the following sentence at the end of the Methods and Findings section of the abstract:*

*“Limitations include data being from a single centre, lack of data on staff vaccinated outside the hospital systems, and staff may have taken up vaccination following data extraction.”*

**Comment 34. Author summary - At this stage, we ask that you reformat your non-technical Author Summary. The Author Summary should immediately follow the Abstract in your revised manuscript. This text is subject to editorial change and should be distinct from the scientific abstract. The summary should be accessible to a wide audience that includes both scientists and non-scientists. Please see our author guidelines for more information: [hyperlink to guidelines]**

*Response: We have now added an author summary immediately following the abstract in our revised manuscript.*

**Comment 35. Please conclude the Introduction with a clear description of the study question or hypothesis.**

*Response: The introduction now concludes with the following statement which details the aim of our work:*

*“To add to the limited evidence base on this important public health issue, particularly in light of the potential requirement for a SARS-CoV-2 booster vaccination for UK HCWS, we sought to determine the effects of demographic factors (including ethnicity), occupational factors and previous COVID-19 on SARS-CoV-2 vaccine uptake in a large multi-ethnic UK healthcare workforce.”*

**Comment 36. The last line of the Introduction section should read, “…and previous COVID-19 infection…”**

*Response: We respectfully disagree that this is how the sentence should read. Our understanding is that COVID-19 is the disease caused by infection with SARS-CoV-2 and our wording reflects this. Therefore, an individual is not infected with COVID-19 but instead infected with SARS-CoV-2 and develops COVID-19 as a result. However, if the editorial team disagree we will make the suggested change.*

**Comment 37. Did your study have a prospective protocol or analysis plan? Please state this (either way) early in the Methods section.**

*Response: . Our study had a prospective analysis plan. This was generated by discussion of the work with all co-authors prior to the data being accessed. However, given that there was no specific funding application prepared for this work and the work did not require ethical review, this was not formally prepared as an independent document. Instead these discussions informed the original draft of the methods section of the paper, which was prepared prior to data extraction.*

*In the data analysis section of the Methods we now state the following:*

*“An analysis plan was generated prospectively through discussion with all co-authors prior to accessing the data. These discussions informed the original draft of the methods section of the manuscript which was prepared prior to data extraction. Dependent variables that may be associated with the outcome (and are routinely collected through ESR) were selected a priori with reference to existing literature. Beyond the collapsing of categories of certain demographic variables (granular ethnicity categories and occupational roles) due to low numbers of HCWs in these categories there were no data-driven changes to the analyses conducted.”*

*We would be happy to provide the editorial team and reviewers with the original draft of the introduction and methods of the paper should they feel this is necessary.*

**Comment 38. If a prospective analysis plan (from your funding proposal, IRB or other ethics committee submission, study protocol, or other planning document written before analyzing the data) was used in designing the study, please include the relevant prospectively written document with your revised manuscript as a Supporting Information file to be published alongside your study, and cite it in the Methods section. A legend for this file should be included at the end of your manuscript…**

*Response: Please refer to the answer to comment 37 above.*

**Comment 39. …If no such document exists, please make sure that the Methods section transparently describes when analyses were planned, and when/why any data-driven changes to analyses took place…**

*Response: In the data analysis section of the Methods we now state the following:*

*“An analysis plan was generated prospectively through discussion with all co-authors prior to accessing the data (although this was not formally prepared as a written document). Dependent variables that may be associated with the outcome (and are routinely collected through ESR) were selected a priori with reference to existing literature. Beyond the collapsing of categories of certain demographic variables (granular ethnicity categories and occupational roles) due to low numbers of HCWs in these categories there were no data-driven changes to the analyses conducted.”*

**Comment 40. …In either case, changes in the analysis-- including those made in response to peer review comments-- should be identified as such in the Methods section of the paper, with rationale.**

*Response: We have now included this information in the Methods section of the manuscript (please see response to comment 39).*

**Comment 41. Please ensure that the study is reported according to the STROBE guideline, and include the completed STROBE checklist as Supporting Information. Please add the following statement, or similar, to the Methods: "This study is reported as per the Strengthening the Reporting of Observational Studies in Epidemiology (STROBE) guideline (S1 Checklist)." The STROBE guideline can be found here: [hyperlink to STROBE guideline]**

*Response: We have ensured that the study is reported according to STROBE guideline for cross sectional studies and have attached the STROBE checklist.*

**Comment 42. In the statistical analysis section, please clearly state all the adjustment variables used.**

*Response: We have now included further details to help clarify the adjustment variables in the statistical analysis section. This reads:*

*“We used logistic regression to evaluate the univariable association of age, sex, ethnicity, deprivation, occupation, SARS-CoV-2 serology and PCR results and the reason given for any COVID-19 related work absences (including symptomatic infection, household infection or pregnancy) with SARS-CoV-2 vaccine uptake and presented the results as odds ratios (ORs) with 95% confidence intervals (95%CIs). We also used multivariable logistic regression to determine adjusted odds ratios (aORs) and 95%CIs after adjustment for age, sex, ethnicity, deprivation, occupation, SARS-CoV-2 serology and PCR results and the reason given for any COVID-19 related work absences.”*

**Comment 43. In statistical methods, please refer to any post-hoc corrections to correct for multiple comparisons during your statistical analyses. If these were not performed, please justify the reasons.**

*Response: We did not employ post-hoc corrections for multiple comparisons. Our aim was to conduct an exploratory analysis to determine factors that might be associated with SARS-CoV-2 vaccine uptake in healthcare workers (at the time of submission we were the first to examine vaccine uptake in a healthcare worker population although surveys of vaccine intention and uptake in healthcare workers have been published since and report comparable results) therefore we did not want to disregard potentially important findings on the basis that p values did not meet a, potentially overly conservative, threshold (i.e. type II error). Instead we chose to focus on effect sizes, presenting odds ratios together with their 95% confidence intervals which allow readers to draw conclusions about the significance of an individual finding.*

*We would note that had we used a Bonferroni correction (regarded as one of the most conservative corrections for multiple comparisons), the variables that we have determined to be important predictors of vaccine uptake (which are covered in detail in the discussion) would not change as the results discussed as being significant have associated p values <0.001 which is below that of the Bonferroni corrected alpha level.*

*If the editorial team feel that the analysis or its interpretation would be improved by applying a Bonferroni correction then we would be happy to do so.*

**Comment 44. In the methods section, where appropriate, please consistently provide both 95% CIs and p values in the text and tables.**

*Response: We have now provided a p value wherever we have presented a 95%CI in the Results section. We do not present any 95%CIs or p values in the Methods section.*

**Comment 45. Please specify the statistical test used to derive the p values.**

*Response: All of the variables in the analysis are categorical and comparison of the unvaccinated and vaccinated groups (outside of the logistic regression analyses) is achieved by using chi-square tests.*

*We have now included a sentence in the Methods section which reads:*

*“All variables were categorical and were summarised as count and percentage. We tested differences between vaccinated and unvaccinated cohorts using chi-squared tests.”*

**Comment 46. Please provide abbreviations in Tables e.g. OR, aOR**

*Response: We have now provided footnotes explaining abbreviations at the bottom of each table. For example the footnote under table 2 reads:*

*“aOR – adjusted odds ratio (adjusted for all variables in the table), HCA – healthcare assistant, IMD – index of multiple deprivation, OR – odds ratio, PCR – polymerase chain reaction, SARS-CoV-2 – severe acute respiratory syndrome coronavirus-2, COVID-19 – coronavirus disease 2019”*

**Comment 47. Discussion section, paragraph 2, line 2, please replace “reduced” with “lower.”**

*Response: We have made the suggested change.*

**Comment 48. Please use the "Vancouver" style for reference formatting and see our website for other reference guidelines [hyperlink to referencing guidelines]**

*Response: We have updated our referencing style to bring it in line with the guidelines.*

**Comment 49. Please use the PLOS Medicine style reference call outs throughout the text, noting the absence of spaces within the square brackets, e.g., "... approved [2,3]."**

*Response: We have updated our reference call outs to square brackets without spaces.*

**Comment 50. Please ensure that journal name abbreviations consistently match those found in the National Center for Biotechnology Information (NCBI) databases. [hyperlink to referencing guidelines]**

*Response: We have now used the suggested journal abbreviations in our bibliography.*

**Comment 50. Please ensure that six names appear before et al.**

*Response: We have now ensured that 6 names appear before et al. in the bibliography.*

**Comment 51. Please include line numbers in the next draft**

*Response: We have now included line numbers in the revised manuscript.*

**Response to Reviewer comments**

**Reviewer 1**

**Comment 1. "Association of demographic and occupational factors with SARS-CoV-2 vaccine uptake in a multi-ethnic UK healthcare workforce: a rapid real-world analysis" studies vaccine uptake of almost 20,000 healthcare workers (HCWs) within a large UK hospital workforce (University Hospitals of Leicester NHS Trust).**

*Response: and for providing an accurate description of our study.*

**Comment 2. Ethnic minority status, younger age, female sex, increasing deprivation and previous confirmed COVID-19 were all found to negatively correlate with vaccine uptake, from adjusted logistic regression modelling.**

*Response: Thank you for highlighting the important results of our analysis.*

**Comment 3. The analysis is relatively straightforward, and the subject matter of urgent interest.**

*Response:* *It was our intention not to over-complicate the analysis in order to make the paper accessible and easily understandable. We also thank Reviewer 1 for recognising the importance of the subject matter and therefore the implications of our findings.*  **Comment 4. The data extends only up to the 3rd of February (Figure 1), whereas some of the vaccination hubs were established only on the 15th of January.**

*Response:* *It is correct that all data were extracted on the 3^rd^ of February 2021 and that the third (of three) vaccination hubs was set up on the 15^th^ January 2021.*

**Comment 5. As such, the study appears to cover a relatively short vaccination period. It might be considered to extend the analysis period if possible, or to discuss mitigating factors (e.g. negligible vaccine uptake after Feb 3).**

*Response: In addressing this comment it is important to understand that although a vaccination hub was established at each of the three University Hospitals of Leicester NHS trust (UHL) sites, any member of staff could be vaccinated at any vaccine hub (as opposed to hospital workers being restricted to receiving vaccination at the hub located at their particular place of work).*

*Whilst we agree that it is likely that some staff were vaccinated after data extraction on the 3^rd^ of February. We felt that, given the clear decrease in total number of staff vaccinated per week (shown in Figure 1), that the vast majority of those who would accept vaccination had already done so at the point of data extraction. We would also make the point that at the time the analysis was conducted we felt it was in the scientific and public interest to release these data urgently so that appropriate adjustments/interventions could be made in order to enhance vaccine uptake in the groups identified within our analysis.*

*Furthermore, our results are broadly similar to a recent UK nationwide survey study of vaccine intention in healthcare workers[1] and with two studies of SARS-CoV-2 vaccine uptake in UK healthcare workers[2, 3] which is reassuring in terms of any effect the time of data extraction may have had on our results.*

*We acknowledge the possibility of staff taking up vaccine after data extraction as a limitation in our discussion. We have modified this in our revision to highlight the similarity of our results to the studies mentioned above. It reads:*

*“We cannot predict if HCWs who are currently unvaccinated will take up vaccination in the future, however the numbers of staff taking up the vaccine over time are falling, implying that most who will accept vaccination have already done so. Furthermore, the predictors of vaccine uptake identified in our surveillance are similar to a recent UK wide survey study of SARS-CoV-2 vaccine hesitancy in HCWs,[1] and two UK based studies on SARS-CoV-2 vaccine uptake[2, 3]providing reassurance about the generalisability of our results.”*

*As regards the possibility of extending the analysis period, this would introduce significant delay in publishing our findings, which Reviewer 1 has kindly acknowledged as being of urgent interest - particularly in light of current discussions around mandating vaccination for healthcare workers and the potential SARS-CoV-2 booster vaccination programme for UK healthcare workers. For reasons given above we believe it is unlikely that predictors of vaccine uptake have changed significantly and therefore do not feel extending the analysis period is necessary.* **Comment 6. In Page 5, it is stated that vaccination was performed initially with the Pfizer-BioNTech vaccine, and subsequently with the Oxford-AstraZeneca vaccine.**

*Response: This is an accurate reflection of the vaccine programme described in the manuscript.*

**Comment 7. It might be clarified as to how the vaccines were assigned/whether HCWs were given a choice of which vaccine to take, and whether this might have influenced uptake.**

*Response: Healthcare workers were not given a choice as to which vaccine they received. We agree it is possible that this could influence uptake, however, we would point out that our analysis pre-dates the well-publicised concerns around the risk of thrombosis following administration of the Oxford-AstraZeneca vaccine (and the subsequent decision by the JCVI to advise that adults aged < 30 are offered an alternative vaccine).[4]*

*Furthermore, it is likely that the same processes (i.e. a lack of choice about which vaccine is received) are replicated across healthcare settings in the UK and any effect this might have on the results is unlikely to affect their generalisability.*

*In order to provide clarity we have added to the relevant section of the methods section which now reads:*

*“UHL began vaccinating staff against SARS-CoV-2 on 12^th^ December 2020, initially using the BNT162b2 mRNA (Pfizer-BioNTech)[5] and subsequently, from 8^th^ January 2021, also with the ChAdOx1 nCoV-19 (Oxford-AstraZeneca)[6] COVID-19 vaccines. Staff were not given a choice of which vaccine they were offered.”*

*We have also added the following to the limitations section of the Discussion:*

*“We are unable to determine which vaccine (Pfizer-BioNTech or Oxford-AstraZeneca) was offered to HCWs, only whether or not they were vaccinated. Therefore, we are unable to determine whether vaccine uptake is associated with the particular vaccine that is offered. This might form a focus of future studies.”*

**Comment 8. In Page 5, it is stated that "Following a short period during which vaccination was limited to those determined to be at highest risk of severe COVID-19, vaccinations were made available to all staff on an ongoing basis". The exact date when vaccination was made available to all, would appear to be very useful in considering the following analysis, and might be stated.**

*Response: We agree that the date that vaccination was made available to all should be included. We have now clarified the timeline of the vaccine rollout in the relevant section of the methods which reads:*

*“At the launch of the vaccine programme, priority was given to those who were ‘clinically extremely vulnerable’ (i.e. those at highest risk of severe COVID-19), were working in a high exposure areas or working with the most vulnerable patient groups. Over the following weeks, this was extended to all staff over 50 years of age or with 2 or more vulnerability factors.[26] Between 7^th^ and 20^th^ January 2021 all remaining patient facing staff were invited to attend. By the end of this period, there was frequently surplus capacity and invitations were extended to any registered health and social care worker in the region (i.e. capacity was such that vaccinations were offered even to those outside the acute hospital trust).”*

**Comment 9. In Page 5, it is stated that vaccination appointments had to be booked. It might be stated whether there were ever any scheduling issues (i.e. under-capacity) thoughout the studied period, such that the vaccination of a willing HCW might have had to be postponed.**

*Response: Vaccine hubs at UHL were well resourced and, to our knowledge, there were not any scheduling issues during the studied period. Furthermore, as capacity increased in early January ‘drop-in sessions’ were advertised to staff. Therefore any willing healthcare worker would have had access to vaccine.*

*We have clarified this in the relevant section of the Methods where we state:*

*“Vaccine hubs at UHL were well resourced and, to our knowledge, there were no instances during the study period of vaccination appointments being postponed due to a lack of capacity. Furthermore, as vaccination capacity increased with the addition of vaccine hubs, drop-in sessions were made available to staff”*

**Comment 10. In Page 10, it is stated that "To our knowledge, we are the first to provide real-life data demonstrating that SARS-CoV-2 vaccine uptake is reduced in ethnic minority HCWs"; however, vaccine hesitancy amongst ethnic minorities appears to be relatively well-known (e.g. from "Coronavirus and vaccination rates in people aged 70 years and over by socio-demographic characteristic, England: 8 December 2020 to 11 March 2021", from the Office for National Statistics). Such claims might thus be moderated.**

*Response: The Office for National Statistics (ONS) document kindly highlighted by the reviewer does indeed indicate that vaccine uptake might be lower in ethnic minority groups compared to those of White British ethnicity. However, in contrast to our analysis of healthcare workers, this is in a general population cohort aged 70 and above. Similar findings to the ONS report were demonstrated by the OpenSAFELY collaborative[7] which we mention in the discussion of our manuscript immediately after the section quoted by the reviewer.*

*At the time of submitting the original manuscript, to our knowledge, we were the first investigators to examine SARS-CoV-2 vaccine uptake in a healthcare worker population. However, in the intervening period between our original submission and the offer to revise the manuscript, two studies (both primarily focussed on vaccine effectiveness) have published data on vaccine uptake in UK healthcare workers.[2, 3] It should be noted that one of these studies is limited by a small sample size and the fact that it was conducted in a specialist orthopaedic hospital and the other is a consented cohort study which, when determining SARS-CoV-2 vaccine uptake, may suffer from self-selection bias (factors that may affect the generalisability of the findings of these two studies).*

*For these reasons, together with the fact that our cohort represent the entire workforce of one of the largest and most ethnically diverse hospital trusts in the UK, we feel our work represents a major addition to the limited existing literature on healthcare worker SARS-CoV-2 vaccine uptake and contains findings that are of urgent public health interest (and this is acknowledged by the reviewers).*

*In light of the above we have now moderated the claim referred to by Reviewer 1. The relevant section of the manuscript now reads:*

*“We provide real-life observational data from an entire hospital workforce demonstrating that SARS-CoV-2 vaccine uptake is lower in ethnic minority HCWs than those of White ethnicity. This finding aligns with the limited number of published studies conducted on vaccine uptake in HCWs to date[2, 3] as well as recent survey studies on SARS-CoV-2 vaccine hesitancy in HCWs in the UK and USA.[1, 8] It also adds significant weight to emerging data in the general population which also suggests reduced uptake in ethnic minority groups. Our findings closely align with this population level data as we also demonstrate that those of Black ethnicity were least likely to take up vaccination and that, amongst South Asian ethnic groups, those of Pakistani and Bangladeshi ethnicity were less likely to take up SARS-CoV-2 vaccination than those of Indian ethnicity[7].”*

**Comment 11. It might be considered to provide figures for vaccination over time (as in Figure 1), for other major demographic/risk factors (e.g. age, sex, IMD status, etc)**

*Response: We agree that some readers may wish to see vaccination rate stratified by other demographic characteristics as suggested by the reviewer. However, we are keen not to draw focus from our findings relating to ethnicity in the main analysis (one of the key strengths of our study is the ethnic diversity of the cohort).* *We have, therefore, prepared figures showing vaccination rates stratified by age, sex and index of multiple deprivation (IMD) quintile as suggested by the reviewer, but have made these available as supplementary figures 2a, 2b and 2c (uploaded together with the revised manuscript).*

*We would, however, move the figures to the main body of the manuscript at the editors discretion.* **Comment 12. While some speculation about reduced vaccine uptake had been attempted (e.g. "limited access to trust communications" for locum doctors), it would be of interest to briefly survey reasons for declining vaccination if possible, including on possible intent for future vaccination.**

*Response: We agree that establishing the underlying mechanisms behind reduced SARS-CoV-2 vaccine uptake in the demographic groups we have identified as being more likely to be unvaccinated is critical, particularly for informing interventions aimed at improving vaccine uptake. Indeed, recent work from some of the co-authors of this paper attempts to determine the underlying reasons behind vaccine hesitancy in ethnic minority healthcare workers in the UK using data from a nationwide cohort study.[9]*

*However, we feel that surveying UHL staff is beyond the scope of the current work. To do so would not only lead to significant delay in the publication of important data but, as discussed above, similar work has already been performed on a national scale. Furthermore, the data were collected for a specific purpose i.e. an audit on vaccine uptake. Inclusion of a survey into this project was not planned and would require review by a research ethics committee which would further delay publication.*

**Reviewer 2


Comment 1. This is an important paper that provides useful insights for implementing vaccination programs in the UK.**

*Response: Thank you for recognising the value of our work. We agree that our paper contains information that is directly relevant to policymakers and those designing SARS-CoV-2 vaccine programmes. We believe our work is particularly relevant at present with the announcement of SARS-CoV-2 booster vaccination for healthcare workers in the UK.*

**Comment 2. I have only few comments for this manuscript:**

*Response: Thank you. We will address each of these comments in turn below.*

**Comment 3. In the introduction, I would like to see more background about the situation of vaccination among HW in other countries.**

*Response: We agree with the reviewer that it would be interesting to compare our results to studies conducted in other countries. However, to our knowledge, there are limited data on SARS-CoV-2 vaccine uptake in HCWs from outside the UK.*

*We have included a short sentence in the introduction that reads:*

*“Data from outside the UK are limited, however, an Israeli study reported SARS-CoV-2 vaccine uptake in HCWs to be 90% but did not attempt to determine factors associated with uptake.[10]”*

**Comment 4. In the discussion, Indeed, HW should be provided the vaccine freely so it is hard to conclude that there are disparities here. Did this issue occur in the previous epidemic?**

*Response: The reviewer raises an interesting point about access to vaccination.*

*In considering vaccine hesitancy, the World Health Organisation has proposed the ‘3C model’ which relates to the 3 main factors that influence vaccine uptake – Convenience (vaccine access), Confidence (trust - in vaccines generally, in their efficacy, in those providing the vaccine, and in those creating vaccine policy), and Complacency (perceived risk of vaccine-related disease).[1, 11]*

*With reference to the 3C model, we agree with Reviewer 2 that for healthcare workers in our analysis who were freely given access to SARS-CoV-2 vaccine at a time of their convenience, and were delivered regular electronic and verbal reminders to book an appointment for vaccination; the ‘Convenience’ element of the 3C model is unlikely to be a major contributor to the differences seen in vaccine uptake by demographic groups. Though it does seem pertinent to mention that in our discussion we highlight that, due to the nature of their work, some occupational groups (e.g. estates and facilities staff) might not have the same access to electronic reminders to book vaccination appointments (compared to, for example, clinical staff) and it is possible this could drive some of differences in uptake between these groups.*

*Nonetheless, it is likely that for the majority of healthcare workers, drivers behind a lower vaccine uptake in particular demographic groups relate to the ‘Confidence’ and ‘Complacency’ factors. Indeed, we make a similar point regarding the drivers behind vaccine hesitancy in relation to ethnicity in our discussion, where we state:*

*“Evidence on the specific barriers to COVID-19 vaccination in ethnic minority groups is limited[12]. However, when vaccine uptake is considered more broadly, factors such as a lack of trust in the government or in healthcare systems (e.g. due to unethical and non-ethnically heterogenous research practices in vaccine studies or structural and institutional racism), a lower perception of the risk of COVID-19 or a higher perception of the risk of side effects from vaccination and other sociodemographic factors interrelated to ethnicity (educational level, socioeconomic status and religion) have all been suggested as barriers to vaccine uptake.[12-15] Additionally, a recent nationwide survey study of UK HCWs has determined that trust in employer and belief in COVID-19 conspiracy theories are important factors in predicting SARS-CoV-2 vaccine hesitancy[1].”*

*We also discuss drivers behind the differences seen between older and younger healthcare workers in the following section of the discussion:*

*“In accordance with previous studies,[1, 8] younger healthcare workers were also less likely to be vaccinated than their older colleagues. A likely explanation for this finding is a reduced perception of personal risk of adverse outcome from COVID-19. However, alongside the obvious greater risk of transmitting infection to more vulnerable individuals, long-term sequelae of COVID-19 (termed ‘long-COVID’) which may cause significant morbidity, has been demonstrated to be prevalent even in a young ‘low-risk’ population,[16] suggesting that this cohort may still derive significant personal benefit from SARS-CoV-2 vaccination.”*

*It is also worth noting that our results (specifically with reference to the disparities mentioned by the reviewer) are in line with other studies in UK HCWs, that have also demonstrated differences in vaccine uptake by ethnic group, levels of deprivation, occupational group and gender[2, 3].*

*With respect to the Reviewer’s comment regarding vaccine uptake in previous pandemics, there is evidence to suggest that ethnic and socioeconomic disparities in vaccine uptake existed during the H1N1 influenza pandemic[17, 18]. We have clarified this with a sentence in the discussion that reads:*

*“Ethnic disparities in vaccine uptake are not unique to COVID-19, as differential vaccine uptake was also seen during the 2009 H1N1 influenza pandemic.[17, 18]”*

**Comment 5. Please provide more implications of this study.**

*Response: Our study identifies demographic and occupational characteristics that may be associated with refusal or delay in acceptance of SARS-CoV-2 vaccination. In determining these factors we have highlighted potential ‘at risk’ groups that should be targeted for interventions in overcoming vaccine hesitancy and in whom the drivers behind a lack of vaccine uptake should be urgently determined. We feel we make this point clearly in the last paragraph of the discussion where we state:*

*“In summary, we have found that in a population of UK HCWs, those from ethnic minority groups and from more deprived areas, as well as younger females and those from particular occupational groups are less likely to take up SARS-CoV-2 vaccination. These findings have major implications for the effective ongoing delivery of SARS-CoV-2 vaccination programmes including the planned booster vaccinations, both in HCWs and in the wider population. Urgent actions should be taken to boost vaccine uptake in the identified groups and prevent the disparities caused by the COVID-19 pandemic from being allowed to widen further.”*

*We also specifically mention the implications of our findings that relate to ethnic differences in vaccine uptake:*

*“Regardless of the underlying mechanisms, these findings give significant cause for concern, as ethnic minority groups (especially those working in healthcare) are at higher risk of infection with SARS-CoV-2 and adverse outcome from COVID-19, yet are not taking up this critical preventative intervention[6, 19-21]. Previous work, including by the UK SAGE, suggests that interventions aimed at overcoming barriers to vaccination in ethnic minority groups might involve; multilingual, non-stigmatising vaccine endorsements from trusted sources (including information aimed at overcoming religious concerns about the vaccine) and community engagement (utilising trusted sources, e.g. general practitioners, within local communities to respond to concerns about vaccine safety and effectiveness).[1, 12, 22] Implementing such interventions urgently is arguably of higher priority in HCWs than in the general population as HCWs represent an important source of health information for ethnic minority communities[12].”*

*One finding that we did not concentrate on in our original submission was the lower vaccine uptake in pregnant HCWs. This finding has important implications for policymakers and we have now added a paragraph to our discussion that reads:*

*Though numbers of pregnant HCWs in our analysis were small, we found that pregnancy was associated with lower odds of being vaccinated. Pregnant HCWs have previously been shown to be SARS-CoV-2 vaccine hesitant[1]. This finding may be a result of the advice given to pregnant women at the start of the vaccine rollout to delay vaccination until after delivery, on the basis that there was a lack of safety data in this cohort. However, there is now accumulating evidence that SARS-CoV-2 vaccination is safe during pregnancy and also that pregnancy increases the risk of severe COVID-19.[23, 24] Therefore, these findings have important implications for targeting catch-up vaccination programmes.*

**Comment 6. Please also provide more details about the vaccination plan of the UK to address the disparities.**

*Response: . We agree with the Reviewer that it is critical that interventions are implemented to address the disparities in vaccine uptake in healthcare workers that we have identified in our analysis.*

*The ethnicity sub-group of the UK Scientific Advisary Group for Emergencies (SAGE), which provides scientific advise to government decision makers during emergencies, prepared an overview of vaccine hesitancy in ethnic minority groups in December 2020. This included evidence on how barriers to vaccination in ethnic minority groups might be overcome. Although we touched upon this in our discussion in the original submission we have now added a short section to our discussion to emphasise this, as well as to highlight some recent research papers that comment on how disparities in SARS-CoV-2 vaccine uptake in the UK might be addressed. This reads:*

*“Previous work, including by the UK Scientific Advisary Group for Emergencies (SAGE), suggests that interventions aimed at overcoming barriers to vaccination in ethnic minority groups might involve; multilingual, non-stigmatising vaccine endorsements from trusted sources (including information aimed at overcoming religious concerns about the vaccine) and community engagement (utilising trusted sources, e.g. general practitioners, within local communities to respond to concerns about vaccine safety and effectiveness).[1, 12, 22] Implementing such interventions urgently is arguably of higher priority in HCWs than in the general population as HCWs represent an important source of health information for ethnic minority communities[12].”* **Reviewer 3**

**Comment 1. Researchers presented the results of cross-sectional surveillance that described SARS-CoV-2 vaccination uptake among more than 19,000 healthcare workers from the University Hospitals of Leicester NHS Trust.**

*Response: Thank you for this comment and for accurately highlighting the design of our surveillance and it’s large sample size.*

**Comment 2. They also determined demographic and socio-economic factors associated with vaccination uptake. The results indicate that vaccine uptake is reduced in ethnic minority healthcare workers, and that is also lower in healthcare workers of younger age, females, and those living in more deprived areas.**

*Response: The reviewer has accurately highlighted our important findings.*

**Comment 3. This is a novel study related to SARS-CoV-2 vaccination uptake; and the manuscript is well written and easy to follow.**

*Response: We are glad that the reviewer found our work to be novel and that the manuscript to be well written and easy to follow. We hope that we have improved upon this further, after the feedback from the expert reviewers, in our revised manuscript.*

**Comment 4. My comments and suggestions are as follows:**

*Response: We will address the comments and suggestions point-by-point below.*

**Comment 5. In the methods section (data collection section in particular), please use subtitles to distinguish independent variables (demographic and socio-economic factors and previous COVID-19 test results from the dependent variable (vaccination uptake).**

*Response: We have now restructured the data collection section of the methods to distinguish the dependent and independent variables. This reads:*

***“Data collection***

***Outcome variable***

*The outcome was uptake of at least one dose of SARS-CoV-2 vaccine. This was established by extracting data from the National Immunisation and Vaccination System (NIVS).*

***Covariates***

*We extracted information concerning age (categorised into groups of ≤30, 31 – 40, 41 – 50, 51 – 60 and ≥61 years old); sex; occupational role (categorised into 7 groups– Supplementary Table 2) and residential postcode from the ESR. We used residential postcode to obtain the Index of Multiple Deprivation (IMD) quintile using an online tool provided by the UK government. IMD is the official measure of relative deprivation for small areas of the UK. [25]*

*We also collected data on self-reported ethnicity (this was categorised into White, South Asian, Black and Other for the main analysis and into a larger number of ethnicity categories [based on the 18 categories used by the UK’s Office for National Statistics] for a more granular subanalysis – Supplementary Table 1). Ethnicity has previously been defined as “the social group a person belongs to, and either identifies with or is identified with by others, as a result of a mix of cultural and other factors including language, diet, religion, ancestry and physical features traditionally associated with race”.[26]*

*We used data from occupational health records to determine the number, date and result of any SARS-CoV-2 polymerase chain reaction (PCR) or anti-SARS-CoV-2 serology tests as well as the reason given for any recorded COVID-19 related absences from work since the start of the pandemic.”*

**Comment 6. How was ethnicity defined (please provide the ethnicity definition and reference)?**

*Response: . We have now provided a definition for ethnicity in the methods section of our manuscript. This reads:*

*“Ethnicity has previously been defined as “the social group a person belongs to, and either identifies with or is identified with by others, as a result of a mix of cultural and other factors including language, diet, religion, ancestry and physical features traditionally associated with race”.[26]”*

**Comment 7. What classification the 15 categories of ethnicity are based on?**

*Response: . The 15 categories for ethnicity are based on the 18 categories used by the UK’s Office for National Statistics. We have now clarified this with the following comment in the methods:*

*“We also collected data on self-reported ethnicity (this was categorised into White, South Asian, Black and Other for the main analysis and into a larger number of ethnicity categories [based on the 18 categories used by the UK’s Office for National Statistics] for a more granular subanalysis – Supplementary Table 1).”*

*As mentioned in response to a comment above, we merged some of the 18 granular ethnicity categories due to low numbers of participants in these groups. This decision is mentioned in the data analysis section of the methods, where we state:*

*“Beyond the collapsing of categories of certain demographic variables (granular ethnicity categories and occupational roles) due to low numbers of HCWs in these categories there were no data-driven changes to the analyses conducted.”*

**Comment 8. In the data analysis section I suggest clarifying that study characteristics are presented based on the vaccination status to help the readers understand what Chi-square test was applied to.**

*Response: . We have now added a sentence to the data analysis section of the methods that states:*

*“We tested differences between vaccinated and unvaccinated cohorts using chi-squared tests.”*

**Comment 9. Please also provide information on where data were missing. Were data missing only for IMD variable as presented in Table 1, or data for other variables were missing, too?**

*Response: Data were missing for 110 values of IMD. There was also data missing for 1 value of vaccination date. We have added a sentence to the results section that reads:*

*“Data were missing for 110 values of IMD and 1 value for date of vaccination.”* **Comment 10. Results - I'd suggest leaving only percentages and leaving out counts, as both counts and percentages are already available in Table 1 (e.g. saying "Of the 19,044 HCW in the cohort, 64.5% had received ....."This will also make the presentation of the results consistent.**

*Response: In the majority of the results section we have removed counts and left percentages as suggested by the reviewer.*

*In the results subsection entitled “Association of prior SARS-CoV-2 infection with vaccination uptake”, we have left the counts for the number of staff tested by PCR and serology. This is because we go on to describe the percentage of those tested that were positive. We feel this is clearer for the reader, however, if the editorial team disagree we are happy to change this.*  **Comment 11. In tables 1, S3, S4, S5 I suggest deleting '%' in each row and stating that all data are presented as n (%).**

*Response: We have now deleted the ‘%’ symbol in each row of tables 1, S3, S4 and S5. We have also made the following statement in the footnote of each of these tables:*

*“All data presented as n(%).”*

**Comment 12. Considering Chi-square was used, the researchers may wish to add a Significance column to table 1 and present p values that reflect results of the Chi-square tests.**

*Response: We have considered including a significance column in table 1, however, we are keen not to make this table too ‘data heavy’ in order to ensure that this descriptive table is clear for the reader. Furthermore, important differences are summarised with chi-square p values in the text and the univariable logistic regression analysis shown in table 2 arguably provides more information than a significance column in Table 1 (as both information on statistical significance of a univariable association and the direction and magnitude of such an association are shown).* *We have, therefore, elected not to make this change but would do so if the editorial team consider it necessary.*  **Comment 12. Table 2 - I suggest defining aOR i.e. what adjustment it pertains to.**

*Response: We agree with Reviewer 3 that it could be made clearer what factors were adjusted for in the multivariable logistic regression analysis. We have now clarified this in the methods where we state:*

*“We also used multivariable logistic regression to determine adjusted odds ratios (aORs) and 95%CIs after adjustment for age, sex, ethnicity, deprivation, occupation, SARS-CoV-2 serology and PCR results and the reason given for any COVID-19 related work absences.”*

*We have also added a footnote to the relevant tables that states:*

*“aOR – adjusted odds ratio (adjusted for all variables in the table)”*

**Comment 13. In the same table, in column 2, all '%'  could be deleted, as it states at the top of the table that all numbers present N vaccinated/N total (%).**

*Response: We have made the suggested change in all relevant tables.*

**Comment 14. In the title of table 2, 'ke' is missing in the word 'uptake'**
*Response: We apologise for this typographical error. We have now corrected this.*

**Comment 15. Discussion: please compare your results with a recently published study of Woolf et al [hyperlink to study]**

*Response: We agree with Reviewer 3 that the discussion would be improved by a comparison of our results to those from the Woolf et al. paper. It should be noted that some of the authors of the current work were also authors on the Woolf et al. paper, indeed the findings of the current work were used to inform the analysis conducted by Woolf et al. (as evidenced by the citation of the pre-print of our manuscript by Woolf et al.).*

*Despite our study having a different outcome measure to the Woolf et al. paper (vaccine uptake as opposed to vaccine hesitancy), it is reassuring to note that our findings (specifically those relating to ethnicity, age, sex, pregnancy and previous COVID-19) are aligned with those of Woolf et al.*

*We now cite the work of Woolf et al. at multiple points throughout the manuscript. Firstly, when we discuss drivers behind COVID-19 vaccination in ethnic minority groups:*

*“Additionally, a recent nationwide survey study of UK HCWs has determined that trust in employer and belief in COVID-19 conspiracy theories are important factors in predicting SARS-CoV-2 vaccine hesitancy[1].”*

*Secondly, when we discuss methods by which barriers to vaccination might be overcome in ethnic minority groups:*

*“Previous work, including by the UK Scientific Advisory Group for Emergencies (SAGE), suggests that interventions aimed at overcoming barriers to vaccination in ethnic minority groups might involve; multilingual, non-stigmatising vaccine endorsements from trusted sources (including information aimed at overcoming religious concerns about the vaccine) and community engagement (utilising trusted sources, e.g. general practitioners, within local communities to respond to concerns about vaccine safety and effectiveness).[1, 12, 22]”*

*Thirdly, when we discuss our findings related to vaccine uptake in pregnant healthcare workers:*

*“Though numbers of pregnant HCWs in our analysis were small we found that pregnancy was associated with lower odds of being vaccinated. Pregnant HCWs have previously been shown to be SARS-CoV-2 vaccine hesitant[1].”*

*Finally, when we discuss the generalisability of our results:*

*“Furthermore, the predictors of vaccine uptake identified in our surveillance are similar to a recent UK wide survey study of SARS-CoV-2 vaccine hesitancy in HCWs,[1] and two UK based studies on SARS-CoV-2 vaccine uptake[2, 3]providing reassurance about the generalisability of our results.”*

**Comment 16. Please also check for other studies published on a similar topic, such as the recent one of Kuter et al [hyperlink to study]**

*Response:* *. We read Kuter et al.’s study, which details results of a survey study investigating attitudes to SARS-CoV-2 vaccination in two large hospitals in Philadelphia (USA). In accordance with our results Kuter et al. determined that younger age and Black ethnicity were risk factors for vaccine hesitancy in a healthcare worker cohort. We have included citations to the work of Kuter et al. when we discuss our findings related to ethnicity:*

*“We provide real-life data from an entire hospital workforce demonstrating that SARS-CoV-2 vaccine uptake is lower in ethnic minority HCWs than those of White ethnicity. This finding aligns with the limited number of published studies conducted on vaccine uptake in HCWs to date[2, 3] as well as recent survey studies on SARS-CoV-2 vaccine hesitancy in HCWs in the UK and USA.[1, 8]”*

We also cite this work when discussing our findings relating to age:

*“In accordance with previous studies,[1, 8] younger healthcare workers were also less likely to be vaccinated than their older colleagues.”*

*It should also be noted that between our original submission and this revision, there have been two studies of vaccine uptake in UK healthcare workers published. A study in a tertiary orthopaedic hospital with a much smaller cohort than that included in the current work[3] and a large national cohort study which, unlike our study and as the authors acknowledge, suffers from the problem of self-selection bias (which explains the high vaccine uptake rate of 90% reported in this study).[2] We have compared our results to these two studies in the discussion of our manuscript.*

**References**

1.	Woolf K, McManus IC, Martin CA, Nellums LB, Guyatt AL, Melbourne C, et al. Ethnic differences in SARS-CoV-2 vaccine hesitancy in United Kingdom healthcare workers: Results from the UK-REACH prospective nationwide cohort study. The Lancet Regional Health – Europe. doi: 10.1016/j.lanepe.2021.100180. PubMed PMID: 100180.

2.	Hall VJ, Foulkes S, Saei A, Andrews N, Oguti B, Charlett A, et al. COVID-19 vaccine coverage in health-care workers in England and effectiveness of BNT162b2 mRNA vaccine against infection (SIREN): a prospective, multicentre, cohort study. The Lancet. 2021;397(10286):1725-35. doi: 10.1016/S0140-6736(21)00790-X.

3.	Azamgarhi T, Hodgkinson M, Shah A, Skinner JA, Hauptmannova I, Briggs TWR, et al. BNT162b2 vaccine uptake and effectiveness in UK healthcare workers – a single centre cohort study. Nature Communications. 2021;12(1):3698. doi: 10.1038/s41467-021-23927-x.

4.	Joint Committee on Vaccination and Immunisation. JCVI statement on use of the AstraZeneca COVID-19 vaccine: 7 April 2021 2021 [cited 2021 11th August]. Available from: <https://www.gov.uk/government/publications/use-of-the-astrazeneca-covid-19-vaccine-jcvi-statement/jcvi-statement-on-use-of-the-astrazeneca-covid-19-vaccine-7-april-2021>.

5.	Polack FP, Thomas SJ, Kitchin N, Absalon J, Gurtman A, Lockhart S, et al. Safety and Efficacy of the BNT162b2 mRNA Covid-19 Vaccine. N Engl J Med. 2020;383(27):2603-15. doi: 10.1056/NEJMoa2034577.

6.	Voysey M, Clemens SAC, Madhi SA, Weckx LY, Folegatti PM, Aley PK, et al. Safety and efficacy of the ChAdOx1 nCoV-19 vaccine (AZD1222) against SARS-CoV-2: an interim analysis of four randomised controlled trials in Brazil, South Africa, and the UK. The Lancet. 2021;397(10269):99-111. doi: 10.1016/S0140-6736(20)32661-1.

7.	MacKenna B, Curtis HJ, Morton CE, Inglesby P, Walker AJ, Morley J, et al. Trends, regional variation, and clinical characteristics of COVID-19 vaccine recipients: a retrospective cohort study in 23.4 million patients using OpenSAFELY. medRxiv. 2021:2021.01.25.21250356. doi: 10.1101/2021.01.25.21250356.

8.	Kuter BJ, Browne S, Momplaisir FM, Feemster KA, Shen AK, Green-McKenzie J, et al. Perspectives on the receipt of a COVID-19 vaccine: A survey of employees in two large hospitals in Philadelphia. Vaccine. 2021;39(12):1693-700. Epub 2021/02/27. doi: 10.1016/j.vaccine.2021.02.029. PubMed PMID: 33632563; PubMed Central PMCID: PMCPMC7885691.

9.	Woolf K, Melbourne C, Bryant L, Guyatt AL, McManus C, Gupta A, et al. The United Kingdom Research study into Ethnicity And COVID-19 outcomes in Healthcare workers (UK-REACH): Protocol for a prospective longitudinal cohort study of healthcare and ancillary workers in UK healthcare settings. medRxiv. 2021:2021.02.23.21251975. doi: 10.1101/2021.02.23.21251975.

10.	Abu Jabal K, Ben-Amram H, Beiruti K, Batheesh Y, Sussan C, Zarka S, et al. Impact of age, ethnicity, sex and prior infection status on immunogenicity following a single dose of the BNT162b2 mRNA COVID-19 vaccine: real-world evidence from healthcare workers, Israel, December 2020 to January 2021. Eurosurveillance. 2021;26(6):2100096. doi: doi:<https://doi.org/10.2807/1560-7917.ES.2021.26.6.2100096>.

11.	MacDonald NE. Vaccine hesitancy: Definition, scope and determinants. Vaccine. 2015;33(34):4161-4. doi: <https://doi.org/10.1016/j.vaccine.2015.04.036>.

12.	Scientific Advisory Group for Emergencies (SAGE - ethnicity sub-group). Factors influencing COVID-19 vaccine uptake among minority ethnic groups, 17 December 2020: UK Government; 2021 [cited 2021 1st February]. Available from: <https://www.gov.uk/government/publications/factors-influencing-covid-19-vaccine-uptake-among-minority-ethnic-groups-17-december-2020>.

13.	Forster AS, Rockliffe L, Chorley AJ, Marlow LAV, Bedford H, Smith SG, et al. Ethnicity-specific factors influencing childhood immunisation decisions among Black and Asian Minority Ethnic groups in the UK: a systematic review of qualitative research. J Epidemiol Community Health. 2017;71(6):544. doi: 10.1136/jech-2016-207366.

14.	Gamble VN. Under the shadow of Tuskegee: African Americans and health care. Am J Public Health. 1997;87(11):1773-8. doi: 10.2105/ajph.87.11.1773. PubMed PMID: 9366634.

15.	Mills M, Rahal C. COVID-19 vaccine deployment: Behaviour, ethics, misinformation   and policy strategies: The Royal Society; 2020 [cited 2021 7th February]. Available from: <https://royalsociety.org/-/media/policy/projects/set-c/set-c-vaccine-deployment.pdf?la=en-%20GB&hash=43073E5429C87FD2674201CA19280A8E>.

16.	Dennis A, Wamil M, Kapur S, Alberts J, Badley AD, Decker GA, et al. Multi-organ impairment in low-risk individuals with long COVID. medRxiv. 2020:2020.10.14.20212555. doi: 10.1101/2020.10.14.20212555.

17.	Burger AE, Reither EN, Mamelund S-E, Lim S. Black-white disparities in 2009 H1N1 vaccination among adults in the United States: A cautionary tale for the COVID-19 pandemic. Vaccine. 2021;39(6):943-51. Epub 2021/01/11. doi: 10.1016/j.vaccine.2020.12.069. PubMed PMID: 33454136.

18.	Ayers CK, Kondo KK, Williams BE, Kansagara D, Advani SM, Smith M, et al. Disparities in H1N1 Vaccination Rates: a Systematic Review and Evidence Synthesis to Inform COVID-19 Vaccination Efforts. J Gen Intern Med. 2021;36(6):1734-45. doi: 10.1007/s11606-021-06715-7.

19.	Sze S, Pan D, Nevill CR, Gray LJ, Martin CA, Nazareth J, et al. Ethnicity and clinical outcomes in COVID-19: A systematic review and meta-analysis. EClinicalMedicine. doi: 10.1016/j.eclinm.2020.100630. PubMed PMID: 100630.

20.	Martin CA, Jenkins DR, Minhas JS, Gray LJ, Tang J, Williams C, et al. Socio-demographic heterogeneity in the prevalence of COVID-19 during lockdown is associated with ethnicity and household size: Results from an observational cohort study. EClinicalMedicine. 2020. doi: 10.1016/j.eclinm.2020.100466. PubMed PMID: 100466.

21.	Martin CA, Patel P, Goss C, Jenkins DR, Price A, Barton L, et al. Demographic and occupational determinants of anti-SARS-CoV-2 IgG seropositivity in hospital staff. Journal of Public Health. 2020. doi: 10.1093/pubmed/fdaa199.

22.	Razai MS, Osama T, McKechnie DGJ, Majeed A. Covid-19 vaccine hesitancy among ethnic minority groups. BMJ. 2021;372:n513. doi: 10.1136/bmj.n513.

23.	UK Government. JCVI issues new advice on COVID-19 vaccination for pregnant women 2021 [cited 2021 May 4th]. Available from: <https://www.gov.uk/government/news/jcvi-issues-new-advice-on-covid-19-vaccination-for-pregnant-women>.

24.	Chervenak FA, McCullough LB, Bornstein E, Johnson L, Katz A, McLeod-Sordjan R, et al. Professionally responsible coronavirus disease 2019 vaccination counseling of obstetrical and gynecologic patients. Am J Obstet Gynecol. 2021;224(5):470-8. doi: 10.1016/j.ajog.2021.01.027.

25.	Ministry of Housing, Communities and Local Government. English Indices of Deprivation 2019 UK2019 [cited 2020 21st June]. Available from: <https://www.gov.uk/government/statistics/english-indices-of-deprivation-2019>.

26.	Bhopal R. Glossary of terms relating to ethnicity and race: for reflection and debate. J Epidemiol Community Health. 2004;58(6):441. doi: 10.1136/jech.2003.013466.
