# Supplementary material for: SARS-CoV-2 vaccine uptake in a multi-ethnic UK healthcare workforce: A cross-sectional study
Source: PLoS Med. 2021 Nov 5;18(11):e1003823. doi: 10.1371/journal.pmed.1003823 (PMC8570522; doi:10.1371/journal.pmed.1003823)
Supplement: Attachment — Submitted filename: UHL vaccine - response to reviewers 14.09.21.docx [file pmed.1003823.s012.docx]

**Corresponding Author**

Dr. Manish Pareek,

Associate Clinical Professor in Infectious Diseases,

Department of Respiratory Sciences,

Maurice Shock Medical Sciences Building,

University of Leicester, University Road,

Leicester, LE1 9HN, UK

Email: [mp426@le.ac.uk](mailto:mp426@le.ac.uk)

Telephone: 0116 2586952

Dr Beryne Odeny

Associate Editor

*PLOS Medicine*

14^th^ September, 2021

Dear Dr Odeny,

**Authors’ response to reviews**

**Factors associated with SARS-CoV-2 vaccine uptake in a multi-ethnic UK healthcare workforce: A cross-sectional study" (PMEDICINE-D-21-00819R2)**

**Now retitled (at editor’s request): SARS-CoV-2 vaccine uptake in a multi-ethnic UK healthcare workforce: A cross-sectional study**

We thank you for recognising the value of our work and are very grateful that you have elected to proceed with publishing our manuscript.

We are also extremely thankful for the insightful comments made by the Editor and the reviewers.

Please find attached a detailed, point-by-point, response to the remaining comments made by the Editor and the reviewers.

Please note we only plan to respond to the specific comments relating to our manuscript as we responded to much of the content in your email regarding for example, author statements and data availability policies, in our previous submission.

Yours sincerely

**Dr. Manish Pareek**

Associate Clinical Professor in Infectious Diseases

**Response to Editor’s comments**

**Comment 1. Thank you very much for re-submitting your manuscript "Factors associated with SARS-CoV-2 vaccine uptake in a multi-ethnic UK healthcare workforce: A cross-sectional study" (PMEDICINE-D-21-00819R2) for review by PLOS Medicine.**

*Response: Thank you for allowing us to submit a revised version of the manuscript.*

**Comment 2. I have discussed the paper with my colleagues and the academic editor and it was also seen again by xxx reviewers. I am pleased to say that provided the remaining editorial and production issues are dealt with we are planning to accept the paper for publication in the journal.**

*Response: We are very grateful for your thorough review of our work and for the insightful comments made by both the editorial and peer review team. We are delighted that you are planning to accept the paper for publication.*

**Comment 3. The remaining issues that need to be addressed are listed at the end of this email. Any accompanying reviewer attachments can be seen via the link below. Please take these into account before resubmitting your manuscript**

*Response: Thank you. We will address each point in turn below.*

**Comment 4. Please consider revising your title to "SARS-CoV-2 vaccine uptake in a multi-ethnic UK healthcare workforce: A cross-sectional study." Apologies for requesting a second revision**

*Response: We have now revised the title according to your suggestion.*

**Comment 5: Please add the following statement to the Methods: "This study is reported as per the Strengthening the Reporting of Observational Studies in Epidemiology (STROBE) guideline (S1 Checklist)."**

*Response: We have now added this statement to the Methods.*

**Comment 6: In revising the manuscript for further consideration, your revisions should address the specific points made by each reviewer and the editors. Please also check the guidelines for revised papers at [hyperlink to guidelines] for any that apply to your paper.**

*Response: We have provided point-by-point responses to each of the editorial and reviewer comments and have checked the guidelines for revised papers.*

**Comment 7. Thank you for providing your STROBE checklist. Please replace the page numbers with paragraph numbers per section (e.g. "Methods, paragraph 1"), since the page numbers of the final published paper may be different from the page numbers in the current manuscript.**

*Response: We have now modified the STROBE checklist accordingly and have uploaded it alongside our revised manuscript.*

**Comment 8. Thank you for updating your references. Please ensure there is a space between in-text reference call outs and the preceding word, and the full stop is placed after the call out. For example, ".approved [2,3]."**

*Response: Thank you. We have amended the manuscript accordingly.*

**Response to Reviewer comments**

**Reviewer 1**

**Comment 1.** **We thank the authors for addressing our previous comments, and have no further queries.**

*Response: We would like to thank Reviewer 1 for taking the time to thoroughly review our work and for recognising its importance. The manuscript has been improved by the incorporation of suggestions made by the expert reviewers.*

**Reviewer 3**

**Comment 1.** **I thank the researchers for addressing all comments and suggestions raised by the editorial team and reviewers. No further comments.**

*Response: We would like to thank Reviewer 3 for taking the time to thoroughly review our work and for recognising its importance. The manuscript has been improved by the incorporation of suggestions made by the expert reviewers.*
